# Editorial: From crosstalk among cell populations in the microenvironment of bone degenerative diseases to the novel therapeutic approaches

**DOI:** 10.3389/fmed.2026.1812496

**Published:** 2026-03-02

**Authors:** Yori Endo

**Affiliations:** Brigham and Women's Hospital, Harvard Medical School, Boston, MA, United States

**Keywords:** biomaterials, bone microenvironment, cell-cell crosstalk, interverbal disc degeneration, osteoarthritis, osteoporosis, regenerative medicine, single-cell transcriptome

Age-related musculoskeletal degenerative diseases—including osteoarthritis, osteoporosis, and degenerative disorders of the spine and periarticular tissues—are characterized by progressive loss of tissue homeostasis shaped by interactions among stromal lineages, immune cells, vascular and neural elements, and the extracellular matrix. This Research Topic highlights how advances in multi-omic profiling, imaging, computational analysis, and emerging therapeutic platforms can be used to clarify microenvironmental signaling networks and inform more targeted interventions for older adults.

The contributions cluster around four complementary areas: population burden and research mapping; molecular and cellular mechanisms; measurement and clinical assessment; and therapeutic, procedural, and rehabilitation strategies. At the population level, comparative burden analysis underscores the scale of osteoarthritis in aging societies and provides quantitative context for translational prioritization (Han et al.). Bibliometric analyses further characterize how evidence and attention are distributed across subfields, including ankle cartilage repair (Fu et al.) and acupuncture for primary osteoporosis (Yu et al.). While bibliometrics does not establish biological validity or clinical efficacy, it can identify research concentrations, emerging themes, and underexplored intersections that warrant targeted mechanistic and clinical evaluation.

Mechanistically, the Topic emphasizes pathway-level programs that can reshape degenerative microenvironments and intercellular communication. Integrative analysis of bulk and single-cell transcriptomes implicates arachidonic acid metabolism in osteoarthritic chondrocytes, illustrating how multi-omics can generate testable hypotheses around candidate regulatory nodes (Wu et al.). In osteoporosis, a focused synthesis highlights ferroptosis as a potential contributor to bone loss and discusses natural product-oriented therapeutic avenues within this framework (Zhu et al.). Together, these papers reinforce a translational principle: mechanistic programs are most informative when they are measurable in patient material, linked to definable disease stages or phenotypes, and paired with intervention strategies that can be evaluated using reproducible endpoints.

Measurement rigor spanning preclinical structural quantification and patient-centered clinical assessment emerges as a second cross-cutting theme. One methodological contribution examines how micro-CT 3D region-of-interest selection influences lumbar vertebral measures in ovariectomized rats, with implications for comparability across preclinical osteoporosis studies (Xu et al.). Complementing this, clinical work evaluates the ASAS Health Index and associated factors in ankylosing spondylitis within a regional cohort, contributing to efforts to standardize health status assessment in chronic axial disease (Li et al.). These studies underscore that reproducible quantification—structural, functional, and patient-reported—is foundational for benchmarking, cross-study synthesis, and translation of biological insight into clinically meaningful benefit.

Therapeutic and implementation-oriented contributions address non-pharmacologic interventions, perioperative management, and surgical strategy—domains with direct relevance to older adults and real-world delivery constraints. A randomized clinical trial evaluates immediate effects of high-intensity laser therapy for nonspecific neck pain, adding evidence on adjunctive, non-drug pain management approaches (Xie et al.). A focused review synthesizes causes and countermeasures for post-operative swelling after gluteal muscle contracture release and organizes preventive strategies across pre-, intra-, and post-operative phases, emphasizing protocolized care and graded rehabilitation (Ren et al.). In the spine, retrospective analyses compare surgical strategies for thoracolumbar fractures and adult L5 spondylolysis, contributing evidence relevant to procedural selection and outcome optimization (Duan et al.; Dong et al.). Across these clinical domains, outcomes reflect not only structural correction but also tissue responses to injury and repair, processes influenced by age, inflammation, and local cellular programs.

Collectively, this Research Topic supports a systems-level view of musculoskeletal degeneration in which multicellular interactions and microenvironmental constraints shape disease progression and treatment response. Several priorities follow. First, mechanistic studies will be strengthened by designs that connect cell states to spatial context, matrix remodeling, mechanics, and immune–vascular coupling, while remaining anchored to measurable clinical endpoints. Second, pathway-level targets (e.g., lipid signaling and regulated cell death) should be advanced with early attention to biomarker strategies, timing, safety, and stage-specific applicability (Wu et al.; Zhu et al.). Third, standardization of measurement from micro-CT regions to health indices and rehabilitation pathways should be treated as a scientific deliverable that enables cross-study comparison and efficient translation (Xu et al.; Li et al.; Ren et al.). Finally, synthesis-oriented mapping approaches can help prioritize future mechanistic and clinical work in rapidly growing areas (Fu et al.; Yu et al.).

In summary, the Research Topic links microenvironmental biology and analytic advances to therapeutic and procedural considerations that influence patient outcomes. By integrating mechanistic insight with measurement rigor and implementation relevance, this Research Topic helps inform the development and evaluation of interventions aimed at preserving mobility and quality of life in aging populations.

